# Megahertz serial crystallography

**DOI:** 10.1038/s41467-018-06156-7

**Published:** 2018-10-02

**Authors:** Max O. Wiedorn, Dominik Oberthür, Richard Bean, Robin Schubert, Nadine Werner, Brian Abbey, Martin Aepfelbacher, Luigi Adriano, Aschkan Allahgholi, Nasser Al-Qudami, Jakob Andreasson, Steve Aplin, Salah Awel, Kartik Ayyer, Saša Bajt, Imrich Barák, Sadia Bari, Johan Bielecki, Sabine Botha, Djelloul Boukhelef, Wolfgang Brehm, Sandor Brockhauser, Igor Cheviakov, Matthew A. Coleman, Francisco Cruz-Mazo, Cyril Danilevski, Connie Darmanin, R. Bruce Doak, Martin Domaracky, Katerina Dörner, Yang Du, Hans Fangohr, Holger Fleckenstein, Matthias Frank, Petra Fromme, Alfonso M. Gañán-Calvo, Yaroslav Gevorkov, Klaus Giewekemeyer, Helen Mary Ginn, Heinz Graafsma, Rita Graceffa, Dominic Greiffenberg, Lars Gumprecht, Peter Göttlicher, Janos Hajdu, Steffen Hauf, Michael Heymann, Susannah Holmes, Daniel A. Horke, Mark S. Hunter, Siegfried Imlau, Alexander Kaukher, Yoonhee Kim, Alexander Klyuev, Juraj Knoška, Bostjan Kobe, Manuela Kuhn, Christopher Kupitz, Jochen Küpper, Janine Mia Lahey-Rudolph, Torsten Laurus, Karoline Le Cong, Romain Letrun, P. Lourdu Xavier, Luis Maia, Filipe R. N. C. Maia, Valerio Mariani, Marc Messerschmidt, Markus Metz, Davide Mezza, Thomas Michelat, Grant Mills, Diana C. F. Monteiro, Andrew Morgan, Kerstin Mühlig, Anna Munke, Astrid Münnich, Julia Nette, Keith A. Nugent, Theresa Nuguid, Allen M. Orville, Suraj Pandey, Gisel Pena, Pablo Villanueva-Perez, Jennifer Poehlsen, Gianpietro Previtali, Lars Redecke, Winnie Maria Riekehr, Holger Rohde, Adam Round, Tatiana Safenreiter, Iosifina Sarrou, Tokushi Sato, Marius Schmidt, Bernd Schmitt, Robert Schönherr, Joachim Schulz, Jonas A. Sellberg, M. Marvin Seibert, Carolin Seuring, Megan L. Shelby, Robert L. Shoeman, Marcin Sikorski, Alessandro Silenzi, Claudiu A. Stan, Xintian Shi, Stephan Stern, Jola Sztuk-Dambietz, Janusz Szuba, Aleksandra Tolstikova, Martin Trebbin, Ulrich Trunk, Patrik Vagovic, Thomas Ve, Britta Weinhausen, Thomas A. White, Krzysztof Wrona, Chen Xu, Oleksandr Yefanov, Nadia Zatsepin, Jiaguo Zhang, Markus Perbandt, Adrian P. Mancuso, Christian Betzel, Henry Chapman, Anton Barty

**Affiliations:** 1 Center for Free-Electron Laser Science, Deutsches Elektronen-Synchrotron DESY, Notkestrasse 85, 22607 Hamburg, Germany; 2 Department of Physics, Universität Hamburg, Luruper Chaussee 149, 22761 Hamburg, Germany; 3 The Hamburg Center for Ultrafast Imaging, Universität Hamburg, Luruper Chaussee 149, 22761 Hamburg, Germany; 4 European XFEL GmbH, Holzkoppel 4, 22869 Schenefeld, Germany; 5 Institute for Biochemistry and Molecular Biology, Laboratory for Structural Biology of Infection and Inflammation, Universität Hamburg, Notkestrasse 85, 22607 Hamburg, Germany; 6 Integrated Biology Infrastructure Life-Science Facility at the European XFEL (XBI), Holzkoppel 4, 22869 Schenefeld, Germany; 7 Australian Research Council (ARC) Centre of Excellence in Advanced Molecular Imaging, Department of Chemistry and Physics, La Trobe Institute for Molecular Sciences, La Trobe University, Bundoora, VIC 3086 Australia; 8 Institute of Medical Microbiology, Virology and Hygiene, University Medical Center Hamburg-Eppendorf (UKE), 20246 Hamburg, Germany; 9 Deutsches Elektronen-Synchrotron DESY, Notkestrasse 85, 22607 Hamburg, Germany; 10 Laboratory of Molecular Biophysics, Department of Cell and Molecular Biology, Uppsala University, Uppsala, 751 24 Sweden; 11 ELI Beamlines, Institute of Physics of the Czech Academy of Sciences, Na Slovance 2, 182 21 Prague, Czech Republic; 12 Condensed Matter Physics, Department of Physics, Chalmers University of Technology, Gothenburg, 412 96 Sweden; 13 Institute of Molecular Biology, SAS, Dubravska cesta 21, 845 51 Bratislava, Slovakia; 14 Biological Research Centre (BRC), Hungarian Academy of Sciences, Temesvári krt. 62, Szeged, 6726 Hungary; 15 Lawrence Livermore National Laboratory, 7000 East Avenue, Livermore, CA 94550 USA; 16 Depart. Ingeniería Aeroespacial y Mecánica de Fluidos ETSI, Universidad de Sevilla, 41092 Sevilla, Spain; 17 Max Planck Institute for Medical Research, Jahnstr. 29, 69120 Heidelberg, Germany; 18 Engineering and the Environment, University of Southampton, SO17 1BJ Southampton, UK; 19 School of Molecular Sciences and Biodesign Center for Applied Structural Discovery, Arizona State University, Tempe, AZ 85287-1604 USA; 20 Hamburg University of Technology, Vision Systems E-2, Harburger Schloßstr. 20, 21079 Hamburg, Germany; 21 Division of Structural Biology, Headington, Oxford, OX3 7BN UK; 22 Diamond Light Source, Research Complex at Harwell, and University of Oxford, Diamond House, Harwell Science and Innovation Campus, Didcot, Oxfordshire OX11 0DE UK; 23 Mid Sweden University, Holmgatan 10, 85170 Sundsvall, Sweden; 24 Paul Scherrer Institut, Forschungsstrasse 111, 5232 Villigen, Switzerland; 25 Department of Cellular and Molecular Biophysics, Max Planck Institute of Biochemistry, 82152 Martinsried, Germany; 26 Linac Coherent Light Source, SLAC National Accelerator Laboratory, Menlo Park, 94025 CA USA; 27 School of Chemistry and Molecular Biosciences, Institute for Molecular Bioscience and Australian Infectious Diseases Research Centre, University of Queensland, Brisbane, QLD 4072 Australia; 28 Physics Department, University of Wisconsin-Milwaukee, 3135 N. Maryland Ave, Milwaukee, WI 53211 USA; 29 Department of Chemistry, Universität Hamburg, Martin-Luther-King Platz 6, 20146 Hamburg, Germany; 30 Institute of Biochemistry, Center for Structural and Cell Biology in Medicine, University of Lübeck, Ratzeburger Allee 160, 23562 Lübeck, Germany; 31 Max-Planck Institute for the Structure and Dynamics of Matter, Luruper Chaussee 149, 22761 Hamburg, Germany; 32 NERSC, Lawrence Berkeley National Laboratory, Berkeley, 94720 CA USA; 33 Biomedical and X-Ray Physics, Department of Applied Physics, AlbaNova University Center, KTH Royal Institute of Technology, Stockholm, 106 91 Sweden; 34 Physics Department, Rutgers University Newark, Newark, NJ 07102 USA; 35 Department of Chemistry, University at Buffalo, 359 Natural Sciences Complex, Buffalo, NY 14260 USA; 36 Institute of Nanostructure and Solid State Physics, Department of Physics, Universität Hamburg, Luruper Chaussee 149, 22761 Hamburg, Germany; 37 Institute for Glycomics, Griffith University, Southport, QLD 4222 Australia; 38 Department of Physics, Arizona State University, Tempe, AZ 85287 USA

## Abstract

The new European X-ray Free-Electron Laser is the first X-ray free-electron laser capable of delivering X-ray pulses with a megahertz inter-pulse spacing, more than four orders of magnitude higher than previously possible. However, to date, it has been unclear whether it would indeed be possible to measure high-quality diffraction data at megahertz pulse repetition rates. Here, we show that high-quality structures can indeed be obtained using currently available operating conditions at the European XFEL. We present two complete data sets, one from the well-known model system lysozyme and the other from a so far unknown complex of a β-lactamase from *K. pneumoniae* involved in antibiotic resistance. This result opens up megahertz serial femtosecond crystallography (SFX) as a tool for reliable structure determination, substrate screening and the efficient measurement of the evolution and dynamics of molecular structures using megahertz repetition rate pulses available at this new class of X-ray laser source.

## Abstract (web summary)

The new European X-Ray Free-Electron Laser (EuXFEL) is the first XFEL that generates X-ray pulses with a megahertz inter-pulse spacing. Here the authors demonstrate that high-quality and damage-free protein structures can be obtained with the currently available 1.1 MHz repetition rate pulses using lysozyme as a test case and furthermore present a β-lactamase structure.

## Introduction

The development of serial femtosecond crystallography (SFX) using intense femtosecond-duration pulses from X-ray free-electron lasers has opened up new avenues for the measurement of macromolecular structures and macromolecular dynamics. SFX has found particular application for room temperature measurements using micron-sized and smaller protein crystals, time-resolved studies of biomolecular dynamics at physiologically relevant temperatures, and the measurement of radiation-sensitive structures^1–7^. The pressing challenge facing serial crystallography has been efficiently measuring diffraction data from the large number of individual micro- or nanocrystals required for the serial crystallography approach. Now, the new European X-ray Free-Electron Laser (EuXFEL) is the first X-ray free-electron laser capable of delivering X-ray pulses with a megahertz inter-pulse spacing, a peak pulse rate four orders of magnitude higher than previously possible^8^. However, to date, it has been unclear whether it would indeed be possible to measure high-quality structures using an XFEL beam with a microsecond X-ray pulse separation. Here, we show that high-quality structures can indeed be obtained using 1.1 MHz repetition rate pulses from the European XFEL using currently available operating conditions (September 2017 and March 2018, proposal p2012). We present two complete data sets, one from the well-known model system in crystallography, lysozyme and the other from a so far unknown complex of a β-lactamase from *Klebsiella pneumoniae* involved in antibiotic resistance. This result opens up the possibility of SFX structure determination at a far higher rate than previously possible, enabling the efficient measurement of the evolution and dynamics of molecular structures using megahertz repetition rate pulses available at this new class of X-ray laser source.

**Fig. 1:**
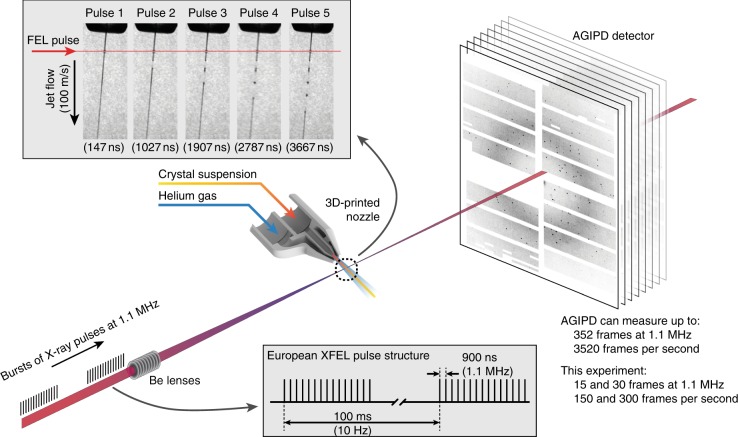
Megahertz serial crystallography. Pulses from the European XFEL were focused on the interaction region using a set of Beryllium lenses. Protein crystals in crystallization solution were introduced into the focused XFEL beam using a liquid jet of 1.8 µm diameter moving at speeds between 50 m/s and 100 m/s. Diffraction from the sample was measured using an AGIPD, which is capable of measuring up to 3520 pulses per second at megahertz frame rates. In-situ jet imaging (inset) showed that the liquid column does explode under the X-ray illumination conditions of this experiment using a jet with a speed of 100 m/s, but that the liquid jet recovered in less than 1 μs to deliver fresh sample in time for arrival of the next X-ray pulse. Images and movies of jets at different speeds are included in the supplementary material

Ultra-short and extremely intense X-ray pulses from XFELs can outrun X-ray-induced damage processes to obtain practically unperturbed structures before the onset of sample explosion^9,10^. "Diffraction before destruction" has enabled the recent development of SFX at FELs using sub-micron-sized crystals at room temperature using doses far exceeding conventional radiation damage limits^11,12^. To date, SFX measurements have been limited by facility pulse repetition rates to measuring at 120 frames per second or 8 ms between pulses^13–15^. The EuXFEL design produces bursts of X-ray pulses at a megahertz repetition rate, repeating at 10 Hz frequency (Fig. 1). At the current EuXFEL, intra-bunch repetition rate of 1.1 MHz the pulse spacing is less than 1 μs, nearly four orders of magnitude shorter than previously available^8^. The decreased time between X-ray pulses enables the EuXFEL to deliver more pulses per second while maintaining the same X-ray peak power, but simultaneously poses several challenges for SFX. Exposed samples must clear the X-ray interaction point in less than 1 μs before the arrival of the next X-ray pulse requiring sample to be delivered four orders of magnitude faster than previously required. Additionally, detecting full-frame diffraction patterns with megahertz pulse repetition rates requires a totally new class of detector. Further complicating matters, the high dose deposited by a single FEL pulse can cause the jet to explode. This creates a void which must also clear the interaction point before the next X-ray pulse arrives. The explosion has been observed to send a shock wave back up the liquid column under certain conditions^16^, while high levels of ionization produced in a small area also create free electrons which can damage as yet unexposed sample. Any of these effects could damage the incoming protein crystals resulting in either modification of the molecular or crystalline structure, possibly preventing structural information to be acquired from diffraction measurements altogether.

We demonstrate here that serial femtosecond crystallography using bursts of megahertz repetition rate X-ray pulses is capable of high-resolution structure determination using high-speed liquid jets as the sample delivery medium and hen egg white lysozyme (HEWL) as a known and well-characterized model system. HEWL is an extremely well-characterized system that crystallizes easily into a range of crystal sizes, making it an excellent system for demonstrating SFX at MHz pulse rates. We further demonstrate that MHz SFX is suitable for structural discovery by determining the structure of a so far unknown complex of a β-lactamase from *K. pneumoniae*. This enzyme belongs to the extended spectrum β-lactamases (ESBLs) that play an important role in emerging multi-antibiotic resistance mechanisms. This class of enzymes is able to hydrolyze the β-lactam ring structure of most prominent antibacterial agents used in medicine and render them ineffective. The constantly evolving resistance to penicillin and penicillin-derived antibiotics is forcing the development of new antibiotics, as particular ESBLs including CTX-M-14 from *K. pneumoniae* are already able to cleave even antibiotics specifically developed against pathogens with high β-lactamase stability including third-generation cephalosporins such as cefotaxime or ceftazidime^17^. These cephalosporins have bulky R1 residues, which means that they no longer fit into the binding pocket of β-lactamases and thus are no longer cleaved by them. The so-called activity-stability compromise for the observed substrate-spectrum-expanding mutations in ESBL describing an enlargement of the binding pocket at the expense of the overall stability of the enzyme^18^ is a suspected cause of inhibition^19^. To obtain structural insights into the molecular basis and spectrum of CTX-M-14 inhibition, we analyzed the complex with the inhibitor avibactam. Furthermore, studying β-lactamase binding is an important demonstration towards both high-throughput substrate screening and future time-resolved diffusion-based SFX experiments in which inhibitor and crystals are mixed on the fly to enable time-resolved structural studies of substrate binding^20^.

## Results

### Megahertz serial crystallography

**Fig. 2:**
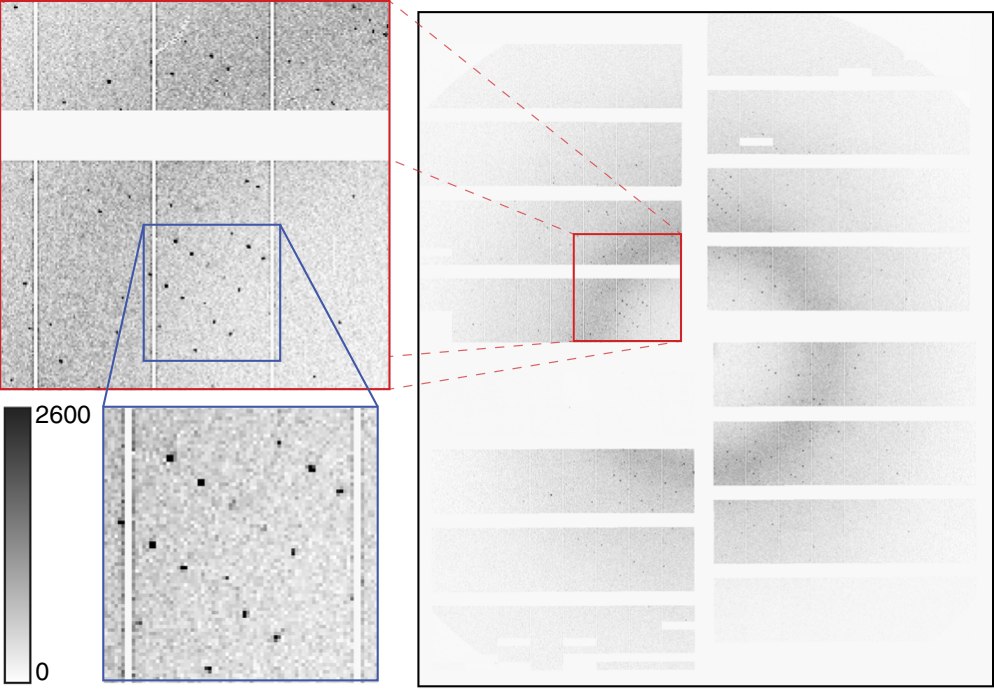
Diffraction pattern from HEWL. Diffraction pattern from a single HEWL microcrystal measured using MHz pulses of 50 fs duration X-rays at 9.3 keV using the AGIPD 1M detector in the SPB/SFX instrument. Dynamic gain switching of the AGIPD detector enables simultaneous low noise and high dynamic range: each pixel has three gain settings which are automatically selected depending on the per-pixel cumulative intensity to simultaneously maximize sensitivity and dynamic range. Image clipped at 2600 counts to show content, full dynamic range of brightest spots extends to 109,000 counts

Our experiment was conducted at the SPB/SFX (single particles, clusters and biomolecules and serial femtosecond crystallography) instrument of the European XFEL^21^. For the HEWL measurements, X-ray pulses with a mean photon energy of 9.3 keV (1.3 Å wavelength), a mean pulse energy of 580 µJ and pulse length of approximately 50 fs duration (derived from the electron bunch length) were focused by beryllium compound refractive lenses into a focal spot of 16 µm diameter full width at half maximum (FWHM) in the SPB/SFX interaction region (Fig. 1). The European XFEL pulse structure for this experiment comprised 15 X-ray pulses at 1.1 MHz repetition rate repeating at 10 Hz, for a total of 150 pulses per second. Microcrystals of HEWL of 6–8 µm size were introduced into the X-ray interaction region in a 1.8 µm diameter liquid jet by a gas dynamic virtual nozzle at speeds of between 50 and 100 m/s. Jet speed was measured using direct imaging in the laboratory under the same conditions as used in the EuXFEL experiment (Table 1). Measurements were made at room temperature and the absorbed dose for each crystal was estimated to be 0.5 MGy using RADDOSE-3D version 2.1^22^ based on an estimated 50% beamline transmission and a 16 µm focal spot size. Diffraction from each X-ray pulse was measured using a 1-megapixel AGIPD (adaptive gain integrating pixel detector) located 0.12 m downstream of the interaction region as shown in Fig. 1. A sample crystal diffraction pattern is shown in Fig. 2 demonstrating the quality of diffraction patterns measured. An image of an HEWL crystal in liquid jet under the same sample delivery conditions used in this experiment is shown in Supplementary Figure 1, which also illustrates how jet speed for crystal solution was directly measured using double exposure illumination.

**Table 1:** Measured jet speeds

Condition	50 m/s	75 m/s	100 m/s	25 m/s
Liquid flow (µL/min)	15	13	13	41
Gas flow (mg/min)	23	50	80	20
**Water**				
Delay time (ns)	200	130	80	~2000
Distance by imaging in lab (µm)	10	10	9	~50
Speed by imaging in lab (m/s)	50	77	110	25
**Lysozyme crystal suspension**				
Delay time (ns)	500	400	200	–
Distance by imaging in lab (µm)	21	31	21	–
Speed by imaging in lab (m/s)	42	78	105	–

**Fig. 3:**
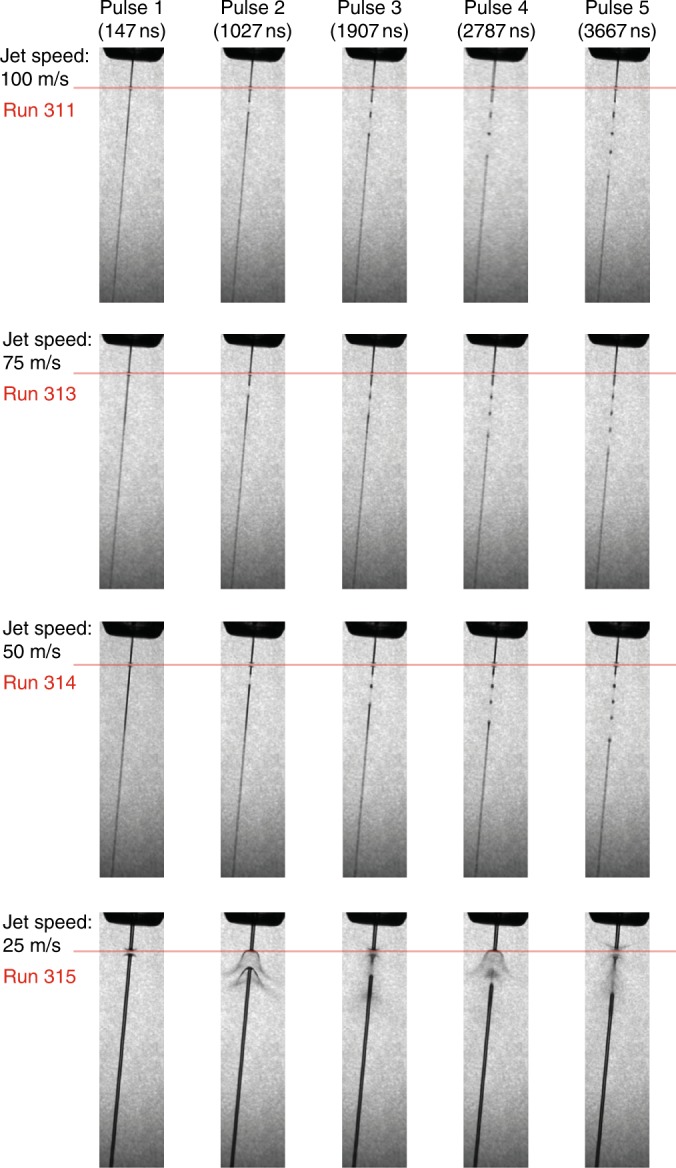
Images of interaction of the EuXFEL liquid jet for the first 5 pulses in a train. Jets in the range of 50–100 m/s recover in time for the next pulse (first three rows), whereas slower jets of the type commonly used at LCLS do not recover in time for the next XFEL pulse at MHz repetition rates (bottom row). The bottom line provides linkage back to the results presented in ref. ^1^. Red line shows the intersection point with X-ray pulses. Images obtained by synchronized laser back illumination. Movies with finer time steps are included as supplementary material

An important consideration is whether data can be collected from any pulse in the EuXFEL pulse train, or only from the first pulse due to jet destruction or crystal damage. Direct imaging of the liquid jet using stroboscopic laser illumination shows that the XFEL pulse initially vaporizes the jet but that the liquid column does indeed recover in time for the next X-ray pulse for jets with a diameter of less than 2 µm and speeds between 50 and 100 m/s, while jets with a speed of 25 m/s do not recover in time (Fig. 3 and Supplementary Movie 1, Supplementary Movie 2, Supplementary Movie 3, Supplementary Movie 4). Imaging reveals that explosion dynamics for jet speeds of between 50 and 100 m/s are qualitatively different from those previously reported^16^, showing a clean break in the liquid stream rather than the rapid expansion shapes reported in ref. ^16^, reflecting the smaller jet size and larger focus compared to previous studies. Results using lower photon energies at FLASH suggest this behaviour will scale to GGy doses expected to be available due to smaller focal spot sizes at the SPB/SFX instrument in the near future^23^. The ratio of focal spot size to jet diameter may also affect explosion dynamics and will be the subject of future studies when smaller focal spot sizes become available.

### Lysozyme reference data

**Fig. 4:**
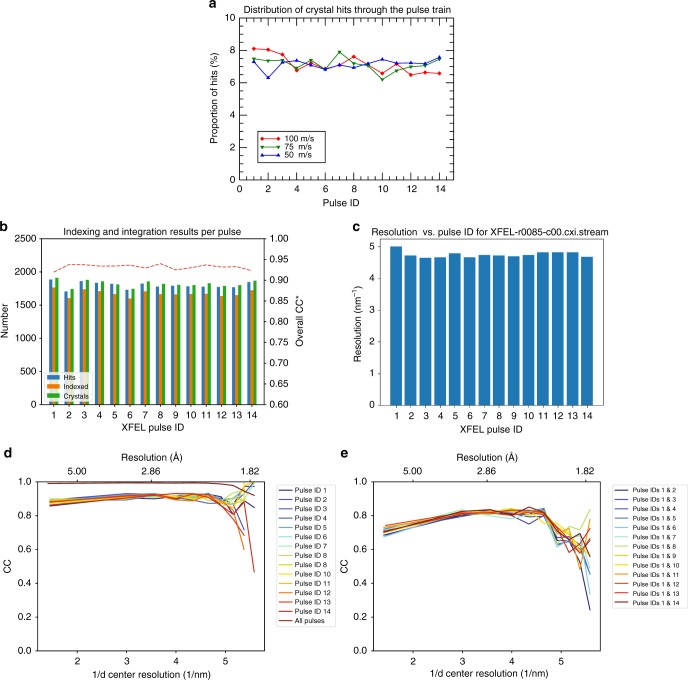
HEWL diffraction was measured on all pulses in the pulse train. **a** Hit fraction as a function of pulse number indicates that crystals are hit randomly on any pulse within the MHz EuXFEL pulse train, and not only on the first pulse in the pulse train. **b** Indexable lattices were equally distributed among the MHz XFEL pulse trains and no sign of degradation in data quality is observed through the pulse train as measured by the overall CC* for subsets of the data corresponding to each pulse. **c** CrystFEL resolution estimate as a function of X-ray pulse within a train shows no decrease in estimated resolution through the course of the pulse train. **d** CC* for data separated from each pulse indicates similar data quality for each pulse in the pulse train. Merging all pulses produces higher data quality (as expected). **e** Correlation of merged data from the first pulse relative to each subsequent pulse in the pulse train indicates that data are similar on each pulse to the limit of data quality available in this experiment. Both **d** and **e** are generated from the same stream files used for structure determination sorted according to pulse ID

We collected 749,874 diffraction patterns from HEWL crystal solution in 83 min of measurement time at 150 pulses per second, of which 25,193 images (3.4%) were found to contain crystal diffraction as identified by Cheetah^24^. We observed that diffraction from microcrystals could be observed on both the first and subsequent pulses in the XFEL pulse train and that detected crystal “hits” were distributed roughly evenly through the pulse train (Fig. 4). This indicates that the first pulse does not destroy the liquid jet for the rest of the pulse train across the range of jet speeds and X-ray pulse intensities tested. From the identified hits, 24,733 images (95%) could be indexed using the CrystFEL software suite^25,26^ yielding 25,531 indexed crystal lattices for structure determination when allowing for multiple lattices per image. Indexing results further indicate that crystals were equally distributed among pulses in the MHz XFEL pulse train with no obvious signs of degradation in data quality through the pulse train (Fig. 4). Additionally, the CC* data metric is similar for merged data split according to pulse ID, and that the correlation between merged data from the first and subsequent pulses is consistent showing no visible signs of degradation under the conditions of this experiment (Fig. 4).

**Fig. 5:**
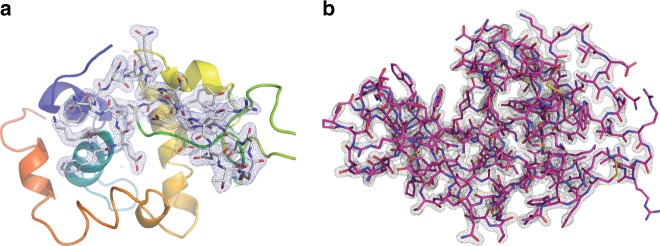
Electron density map for HEWL by MHz SFX. **a** 2Fo-Fc map at 1 sigma overlaid on Fo-Fc map at 3 sigma from molecular replacement using a solvent-free version of the 4ET8 lysozyme structure^2^ as the starting model. **b** Integrity of the measured data is verified by complete rebuilding of the structure from a truncated starting model after removal of residues 1–16 and 40–60 of the polypeptide chain using Autobuild^28^

Merging reflection intensities using the program partialator in CrystFEL produced a data set with an error metric *R*_split_ of 0.105 to 1.8 Å resolution and CC* of 0.995 (Table 2). The structure was determined by molecular replacement using *Phenix*^27^ using a solvent-free version of the 4ET8 SFX lysozyme structure^2^ as the starting model with an *R*_work_/*R*_free_ of 0.151/0.176 to 1.8 Å resolution (Fig. 5a and Table 2). Calculation of a composite simulated annealing omit map and, separately, complete rebuilding of the structure from a truncated starting model using Autobuild^28^ after removal of residues 1–16 and 40–60 of the polypeptide chain indicate that the measured data contain meaningful and sufficient information to rebuild the structure (Fig. 5b and Supplementary Figure 2). No obvious signs of damage are visible at the disulfide bond sites at this dose (Supplementary Figure 3).

**Table 2:** SFX data and refinement statistics

Parameter	Lysozyme	CTX-M-14
Photon energy (mean value)	9300 eV	9150 eV
X-ray focus	15 µm (FWHM)	15 µm (FWHM)
Pulse energy at sample (assuming 50% beamline transmission)	290 µJ	526 µJ
Pulse length	50 fs	50 fs
Space group	P 4_3_ 2_1_ 2	P 3_2_ 2 1
Unit cell		
*a*, *b*, *c*	79.6, 79.6, 38.3 Å	41.8, 41.8, 233.3 Å
*α*, *β*, *γ*	90, 90, 90°	90, 90, 120°
No. of hits/indexed lattices	25,193/25,531	14,445/12,474
No. of unique reflections	12,387 (1171)	27,838 (2715)
Resolution range	21.99–1.76 (1.82–1.76) Å	34.6–1.69 (1.75–1.69) Å
Completeness	99.64% (97.25%)	99.89% (99.45%)
*R*_split_	0.106 (0.446)	0.197 (0.476)
*I*/σ(*I*)	7.36 (2.62)	4.37 (2.30)
CC_1/2_	0.98 (0.79)	0.93 (0.63)
CC*	0.99 (0.94)	0.98 (0.88)
Wilson *B*-factor	26.18 Å^2^	26.80 Å^2^
*R*_Work_	0.157 (0.211)	0.176 (0.27)
*R*_Free_	0.173 (0.218)	0.21 ((0.30)
Rmsd bonds/Rmsd angles	0.010 Å/0.994°	0.008 Å/1.22°
Ramachandran favored	99.21%	98.1%
Ramachandran allowed	0.79%	1.5%
Ramachandran outliers	0.00%	0.4%
Average *B*-factor	30.0 Å^2^	27.6 Å^2^
Macromolecules	28.9 Å^2^	27.1 Å^2^
Ligands	45.8 Å^2^	22.2 Å^2^
Solvent	40.3 Å^2^	37.0 Å^2^
PDB code	6FTR	6GTH
CXIDB data deposition	CXIDB ID-80	CXIDB ID-83

Statistics for the highest-resolution shell are shown in parentheses

### CTX-M-14 β-lactamase

**Fig. 6:**
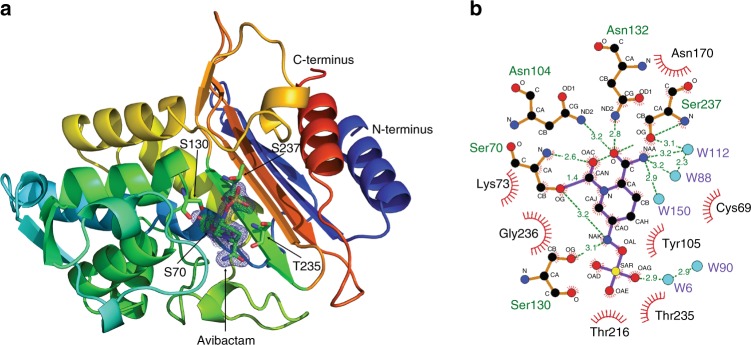
Structure of CTX-M-14 β-lactamase determined by MHz SFX. **a** 2Fo-Fc map at 1 sigma overlaid on Fo-Fc map at 3 sigma around covalently bound avibactam from molecular replacement using a solvent-free version of the 5TWD β-lactamase structure from ref. ^18^ as the starting model. **b** Representation of covalently bound avibactam to OG of Ser70, stabilized by hydrogen bonds and hydrophobic interactions with surrounding amino acids of CTX-M-14. Figure was prepared using Ligplot^53^

Measurements for CTX-M-14 β-lactamase were made in the same manner as for HEWL, except the EuXFEL delivered 300 pulses per second with a mean photon energy of 9.15 keV and a higher mean pulse energy of 1.05 mJ per pulse, giving an absorbed dose of 0.9 MGy using RADDOSE-3D version 2.1^22^. Microcrystals of CTX-M-14 β-lactamase were of 3–8 µm size and delivered using similar jet speeds and diameters as for HEWL. A total of 3,215,616 diffraction pattern were collected from CTX-M-14 collected from which 14,445 (0.4%) were identified as crystal hits by Cheetah^24^, of which 12,474 could be indexed using the CrystFEL software suite^25,26^. Merging reflection intensities using the program partialator in CrystFEL produced a data set with an error metric *R*_split_ of 0.197 and CC* of 0.984 to 1.7 Å resolution (Table 2). A solvent-free version of 5TWD CTX-M-14^18^ was applied to refine the model of CTX-M-14 in complex with avibactam. The SFX data collected to 1.7 Å show a complex with diazabicyclooctane avibactam, covalently bound to OG of Ser70 of the β-lactamase, as also reported similar by King et al.^29^ for other β-lactamases. The crystals are in a space group with only one molecule in the asymmetric unit (AU), an active site fully accessible to solvent, and were soaked with avibactam just before the SFX data collection. The electron density as well as the resulted and refined model are of high quality, without any indication of radiation damage and show avibactam complexed covalently to OG of Ser70 of the β-lactamase (Fig. 6a, b). As CTX-M β-lactamases are known to demonstrate a unique capacity to expand their substrate profile, via active site region amino acid changes, thereby conferring resistance which in turn leads to therapy failure, the obtained CTX-M-14 structure is most useful and complements information already obtained from other β-lactamases^29^. The data will support drug discovery investigations to extend the spectrum of inhibition to a wider range of serine β-lactamases. Experimental procedures applied for crystal preparation, soaking and SFX data collection pave the way for time-resolved SFX experiments applying β-lactamase microcrystals at EuXFEL with different β-lactam antibiotics, such as cefotaxime, to unravel the structural mystery and conformational changes involved in sequential acylation and deacyalation of the β-lactam ring.

## Discussion

The results obtained for HEWL and CTX-M-14 demonstrate that SFX using X-ray pulses with megahertz repetition rates is suitable for high-resolution structure determination using the methods described in this paper under the exposure conditions currently available at the European XFEL. This work was performed with pulse trains of 15 and 30 X-ray pulses delivered in bursts with a 1.1 MHz inter-pulse repetition rate, for a total of 150 and 300 pulses per second—the number of pulses available at the time of the experiment during instrument commissioning. However, the advance to exploiting sub-microsecond inter-pulse spacing demonstrated here is the key to high speed data acquisition using MHz pulse rates. Subsequent experiments have already been able to take advantage of 500 pulses per second^30^, and at the time of writing SFX experiments at the European XFEL are already performed using 1200 pulses per second at 1.1 MHz pulse rates enabling data to be measured at a rate 10 times higher than previously possible at hard X-ray XFELs. 3520 pulses per second is planned to be available for EuXFEL users in 2019 enabling even higher data acquisition rates in the near future. The results presented here demonstrate that SFX using X-ray pulses with sub-microsecond inter-pulse spacing is suitable for high-resolution structure determination, and thus the results scale to even more pulses per train up to and beyond the current AGIPD memory cell limit of 352 pulses per train.

We can therefore look forward to measurements using more pulses per unit time as the number of pulses per train delivered by European XFEL continues to increase. For example, when the number of pulses in the European XFEL pulse train is increased to match the maximum AGIPD detector frame rate of 3520 frames per second, the HEWL data set presented here could be collected in as little as 3.5 min while consuming only 50 μL of crystal solution. Under such conditions the β-lactamase measurements could be completed in only 15 min despite the low hit fraction of 0.4% obtained. Further reduction to less than 1 min per data set consuming only 15 µL of solution should be possible by increasing the hit fraction and reducing dead time through improvements in sample delivery. Moving beyond 352 pulses per train at EuXFEL would require either a new detector able to detect more pulses per pulse train or exploitation of the real-time veto capabilities of AGIPD. Meanwhile, the planned LCLS-II facility promises up to 10^5^–10^6^ equally spaced pulses per second increasing the rate of structure determination even further. In particular, the structural data obtained for the CTX-M14 avibactam complex demonstrates the potential of megahertz SFX for structural discovery at newly available high repetition rate sources, opening up new possibilities for rapid screening for drug targets using on-the-fly substrate mixing, while the potential for rapid data acquisition will facilitate the generation of time-resolved movies of macromolecules in action at physiological temperature.

## Methods

### Sample preparation

Crystals of HEWL were grown by the rapid-mixing batch method^31^. Crystals with sizes of between 6 and 8 µm in diameter were obtained by adding three parts of precipitant (1 M NaCl, 40%(v/v) ethylene glycol, 15%(w/v) PEG 4000, 50 mM acetate buffer pH 3.5 filtered through a 450 nm filter) to one part of HEWL (Sigma–Aldrich; dissolved to 126 mg/mL in 50 mM acetate buffer pH 3.5 and filtered through a 100 nm filter) at 1 °C (ThermoStat C, Eppendorf, Germany). The resulting mixture was immediately subjected to rapid mixing and incubated for 30 min at 1 °C^32^. Crystal sizes were estimated through image analysis by optical microscopy. Crystals were resuspended before injection to yield a homogenous suspension of HEWL microcrystals.

For the CTX-M-14 β-lactamase the gene was cloned into a pRSET A plasmid and transformed into competent *Escherichia coli* BL21DE3 cells (Bl21(DE3) pLyS, Novagen, Schwalbach, Germany). Chromosomal DNA from clinical *K. pneumoniae* DT1 (GenBank CP019077.1) served as a template. The amplicon was cloned into pCR4 and introduced into *E. coli* TOP10 cells (Invitrogen), giving *E. coli* TOP10 x pCR4::blaCTX-M-14. TOP10 x pCR4::blaCTX-M-14 was used to isolate CTX-M14. The primers used to amplify blaCTX-M14 were Prom-CTX-M14-for GCCAAAAGTTATTCTACACTCACT and CTX-M14-rev TTACAGCCCTTCGGCGATG. BL21DE3 cell were grown in LB medium at 37 °C containing 100 µg/mL ampicillin for plasmid selection. Gene expression was induced by supplementation of IPTG (isopropyl β-d-1-thiogalactopyranoside) to a final concentration of 1 mM at an optical density (OD) of 0.7. Cells were harvested 3 h after induction by centrifugation with 4000 × *g* at 4 °C. The cell pellet was resuspended in 20 mM MES pH 6 and sonicated for lysis. Cell debris were separated by centrifugation at 17000 × *g* for 1 h at 4 °C. Supernatant was supplemented by addition of 1 µl DNase and dialyzed overnight against a large volume of 20 mM MES pH 6 at 4 °C. Dialyzed sample was filtered using a 0.2 µm syringe filter and applied onto a cation exchange column (HiTrap SP XL) using a Äkta Pure chromatography system. The column was prequilibrated with 20 mM MES pH 6 and CTX-M-14 was eluted using a gradient over 20 column volumes with 50 mM NaCl, 20 mM MES pH 6. Elution peak was concentrated using a 10 kDa Amicon concentrator to a final CTX-M-14 concentration of 20 mg/mL. CTX-M-14 microcrystals for SFX were produced using a seeding approach. Crystals were grown by sitting drop vapor diffusion at 20 °C overnight mixing 1 µL CTX-M-14 at 20 mg/mL and 1 µL precipitant (40% PEG8000, 200 mM lithium sulfate, 100 mM sodium acetate). Obtained crystals (space group P2_1_2_1_2_1_) were crushed and a seed stock was prepared. To obtain microcrystals the undiluted seed stock was used for batch crystallization setups by mixing volumes of 50% CTX-M-14 with 10% undiluted seed stock and 40% precipitant solution. Resulting microcrystals were centrifuged at 200 × *g* for 5 min and the pellet was crushed using a glass tissue homogenizer. This procedure was repeated 10 times and the supernatant of a final centrifugation step was used for two successive rounds of seed stock preparation, resulting in approximately 1 mL of highly concentrated seed stock that was used for following CTX-M-14 batch crystallization setups. CTX-M-14 microcrystals prepared by this approach grew within 1 h and had a homogeneous size distribution ranging from 3 to 8 µm, scored by light microscopy. Prior to sample loading into the reservoir container the crystal suspension was filtered using a 20 µm gravity flow filter and mixed at this time with avibactam to obtain a final avibactam concentration of 20 mM.

### Fast jets

Delivery of suspensions of crystal solution followed the principle of a gas dynamic virtual nozzle^33–35^ in which a liquid stream is focused and accelerated by the virtual orifice created by a co-propagating helium gas flow. The sample was delivered to the injector using a syringe approach in which a high-pressure liquid chromatography (HPLC) pump (Shimadzu) delivered water to drive the plunger in a sample reservoir, forcing sample through a syringe into the injector lines. The pump delivers a constant flow even at high pressures and thus allows for stable and steady delivery of the sample suspension. The sample flow rate was additionally monitored by a liquid flow meter (Sensirion) located in the water stream between the HPLC pump and the sample syringe/reservoir. Gas flow was controlled using a GP1 gas pressure regulator (Proportion-air) and the flow rate was monitored with a gas flow meter (Bronkhorst). Nozzle tips were produced by three-dimensional (3D) printing^36^ following the design shown in Fig. 1. A 50 µm internal diameter injector sample line was used for improved stability with the crystal sizes used, placing an upper limit on achievable jet speed in this experiment. Three different conditions were chosen for sample delivery spanning the range of 50–100 m/s jet speed, significantly faster than previous jet velocities that were usually below 30 m/s^37^. Jet speeds were estimated during the experiment based on the flow conditions and known geometry of the 3D-printed nozzle, and subsequently verified by high-speed imaging in the laboratory using the same flow conditions as listed in Table 1. Laboratory measurement with both water and HEWL crystal suspension showed similar jet speeds, as reported in Table 1. The speed of the 25 m/s jet was calculated with less precision from movement of the X-ray-induced gaps at EuXFEL. No crystal diffraction data were collected with the 25 m/s jet. For simplicity in the main text and figures we refer to these conditions as jets with a speed of 100, 75, 50 and 25 m/s, values which retain physical meaning but do not over-estimate the stability of the jet speed over time.

Placement of injector nozzles near the XFEL interaction region was achieved using a “nozzle rod” mount provided by the EuXFEL sample environment group, providing the ability to optimize overlap between the focused X-ray beam and the sample-containing liquid jet. Interaction of the jet with the XFEL was imaged using an in-situ microscope with pulsed laser back illumination (Coherent Minilite-II, pulse duration of 3–5 ns for the frequency doubled 532 nm pulse) synchronized to the XFEL pulses similar to the arrangement in ref. ^16^. Jet explosion movies were collected using the higher pulse energy of the β-lactamase measurements.

### SPB/SFX instrument

Experiments were performed at the SPB/SFX instrument at the European XFEL X-ray free-electron laser in September 2017 (HEWL) and April 2018 (CTX-M-14) as part of EuXFEL experiment p2012 using parameters as described in the main text. The size of the focal spot in the interaction region was estimated to be 16 µm ± 4 µm FWHM diameter based on optical imaging of single shots using Ce:YAG screens of various thicknesses (15, 20 and 50 µm). An analysis of the scattered signal on the detector suggests it is possible the actual focal spot was somewhat smaller in size. The liquid jets (described above) were positioned in the interaction region by mounting nozzles on a movable “nozzle rod” which held the jets just above the X-ray focal position and aligned to the X-ray beam using an in-line microscope viewing system. Diffraction from the sample was measured using an AGIPD 1M located 0.12 m downstream of the sample interaction region, with the direct beam passing through a central hole in the detector to a beam stop further downstream.

The AGIPD (Supplementary Figure 4) is a new charge integrating detector built for the European XFEL that is capable of measuring full frames at the EuXFEL pulse repetition rate. The AGIPD is designed to read out in burst mode because the EuXFEL delivers trains of X-ray pulses at MHz repetition rate, repeating at 10 Hz repetition rate. This experiment was performed with 30 pulses per burst at 1.1 MHz repetition rate. The EuXFEL design parameters extend to bursts of up to 2700 pulses at 4.5 MHz repetition rate, and thus each AGIPD pixel contains 352 analog memory cells which can be addressed at MHz repetition rates enabling the AGIPD to measure bursts of up to 352 individual X-ray pulses at MHz repetition rate. Subsequently, all memory cells are read out in the less than 100 ms before arrival of the next burst of X-ray pulses. This enables up to 352 pulses per train to be measured, or when fewer than 352 pulses populate a pulse train allows all pulses to be measured, as is this case here. The maximum frame rate of AGIPD is therefore 3520 frames per second matched to the EuXFEL pulse structure. Each pixel of AGIPD has three gain settings which are automatically selected on a frame-by-frame basis depending on the signal present in each pixel (Supplementary Figure 4). The AGIPD 1M detector used here consists of 16 tiles of 128 × 512 pixels each arranged as shown in Fig. 1 and Supplementary Figures 4 and 5. Calibration of the AGIPD readout requires measurement of the pedestal, gain and gain switching threshold for each of the three gain stages in each memory cell of each pixel. In this experiment the detector readout was initially limited to the first 15 X-ray pulses during instrument commissioning (HEWL), and later 30 pulses (CTX-M-14).

### XFEL data analysis

Experiment progress was monitored online using OnDA^38^ for serial crystallography reading data in real time from the EuXFEL control system Karabo^39^ using the Karabo bridge^40^. Of the 749,874 diffraction patterns collected during HEWL data acquisition runs used for final analysis, 25,193 (3.4%) images were found by Cheetah^24^ to contain crystal diffraction (peakfinder8, minSNR = 8, minADC = 200, minPix = 2, minPeaks = 20). The same procedure was followed for CTX-M-14, except with the peakfinder8 parameters minSNR = 8, minADC = 250, minPix = 1, minPeaks = 20. Data from each AGIPD module was saved into separate files, and thus Cheetah^24^ was updated to match data from each of the 16 separate modules by train and pulse number. This ensured data was processed from the same X-ray pulse even in the presence of missing data frames, for example, if not all modules were present in the saved data for all train and pulse ID combinations. Data were read from uncalibrated (RAW_) data files in European XFEL format, and thus detector calibration was required. See Supplementary Figure 4 for operation of the AGIPD multiple-gain mode. AGIPD calibration was performed in Cheetah as follows: first the memory cell in use for the given Train ID and Pulse ID combination was determined, and then the recorded gain switch level was compared against the gain threshold for that memory cell to determine which gain mode the pixel was in for that particular measurement. The pedestal and gain correction for that memory cell and gain stage was then applied, and a per-memory cell and gain stage bad pixel mask was applied. Bad pixels were identified as statistical outliers in dark data sets and flagged to be ignored. The density of bad pixels across the detector areas used for analysis was 2.5% (Supplementary Figure 5). Calibration constants were obtained using software from both EuXFEL and the AGIPD detector consortium^41^. The output from Cheetah was stored in .cxi format for compatibility with downstream processing. Corrected data frames as well as raw data for both data sets have been deposited in the CXIDB.

### SFX data processing

Indexing was done for both data sets applying CrystFEL v.0.6.3 to peaks found by Cheetah using the indexing packages MOSFLM^42^, DirAx^43^ and asdf^26^. Since detector panel locations were not measured to adequate precision before the experiment, lithium titanate powder diffraction rings were used for rough detector panel alignment followed by fine refinement from HEWL and CTX-M-14 diffraction data using geoptimiser^44^ and Slip-n-slide^45^. Combined with a 1% uncertainty in photon energy and uncertainty in the detector-to-sample distance, final indexing involved an iterative process with refinement of all unknown values using geoptimiser^44^. Indexing of multiple lattices per image sometimes resulted in a higher number of indexed lattices than number of input images. Merging and scaling of the Bragg peaks intensities were performed using partialator program from CrystFEL. To avoid the integration of noise for weakly scattering patterns, reflections were included up to 0.2 nm^−1^ above a conservative resolution estimate for each crystal (--push-res = 0.2) for both HEWL and CTX-M-14. Since the space group of the CTX-M-14 crystals (P 3_2_ 2 1) is merohedral and will exhibit indexing ambiguities, we processed the stream-file using ambigator in CrystFEL^46^ to resolve the indexing ambiguity before scaling and merging. MTZ-files for crystallographic data processing were generated from CrystFEL merged reflection data files using f2mtz of CCP4^47^. Figures of merit were calculated using compare_hkl (Rsplit, CC_1/2_, CC*) and check_hkl (SNR, multiplicity, completeness), both a part of CrystFEL. The distribution of peak intensities and Wilson plot for the HEWL data set also reflect good data quality and dynamic range (Supplementary Figures 6 and 7).

### Structure determination

A solvent-free version of the 4ET8 lysozyme structure^2^ and the solvent-free structure of the 5TWD β-lactamase structure^18^ were used each as a starting model for molecular replacement in Phaser^48^. Due to non-isomorphism of the collected data set with the data set in 4ET8 and 5TWD R_free_-flags were generated randomly using phenix.refine^49^ and the same set of *R*_free_-flags were then used throughout the refinement process. Initial refinement was carried out for both structures using phenix.refine, with all isotropic atomic displacement parameters set to 20 and using simulated annealing. This was followed by iterative cycles of restrained maximum-likelihood refinement using phenix.refine and manual model re-building using COOT^50^. Polygon^51^ and MolProbity^52^ were used for the validation of the final model.

To assess the quality of data we followed two separate approaches. In the first approach, firstly we calculated a composite simulated annealing omit map for the HEWL structure, using phenix.composite_omit_map^27^ (Supplementary Figure 2), and secondly we generated a polyAla-model of the final refined model, truncated residues 1–16 and 40–60 and used AutoBuild^28^ to see whether the final model could be rebuilt correctly and completely starting from just the X-ray-data and the truncated model (Supplementary Figure 2). Figures were generated using PyMOL. For CTX-M-14 the quality of the data and electron density was proven by the clear difference electron density of avibactam complexed in the active site, allowing an unambiguous interpretation of the inhibitor and identification of the covalent bond to OG of Ser70 of the β-lactamase.

### Code availability

The versions of Cheetah and CrystFEL used in this work are available from the respective websites: https://www.desy.de/~barty/cheetah and https://www.desy.de/~twhite/crystfel.

## Electronic supplementary material

Supplementary Information

Description of Additional Supplementary Files

Supplementary Movie 1

Supplementary Movie 2

Supplementary Movie 3

Supplementary Movie 4

## Data Availability

Source data have been deposited with the Coherent X-ray Imaging Databank (CXIDB) with reference number CXIDB-ID-80 (HEWL) and CXIDB-ID-83 (CTX-M-14). Data deposition with CXIDB includes: Raw EuXFEL data files (/raw); Cheetah folder (results and calibrations); Stream files generated by CrystFEL; Detector geometry files; Data calibrated by the European XFEL (/proc). The DOI for the original data at EuXFEL is: https://doi.org/10.22003/XFEL.EU-DATA-002012-00. Structures have been deposited with the Protein Data Bank (PDB) with the accession codes 6FTR (HEWL) and 6GTH (CTX-M-14). Other data are available from the corresponding authors upon reasonable request.
