# Supplementary material for: Megahertz serial crystallography
Source: Nat Commun. 2018 Oct 2;9:4025. doi: 10.1038/s41467-018-06156-7 (PMC6168542; doi:10.1038/s41467-018-06156-7)
Supplement: Supplementary file 1 — Supplementary Information [file 41467_2018_6156_MOESM1_ESM.pdf]

Supplemental information

Megahertz serial crystallography

Wiedorn et al.

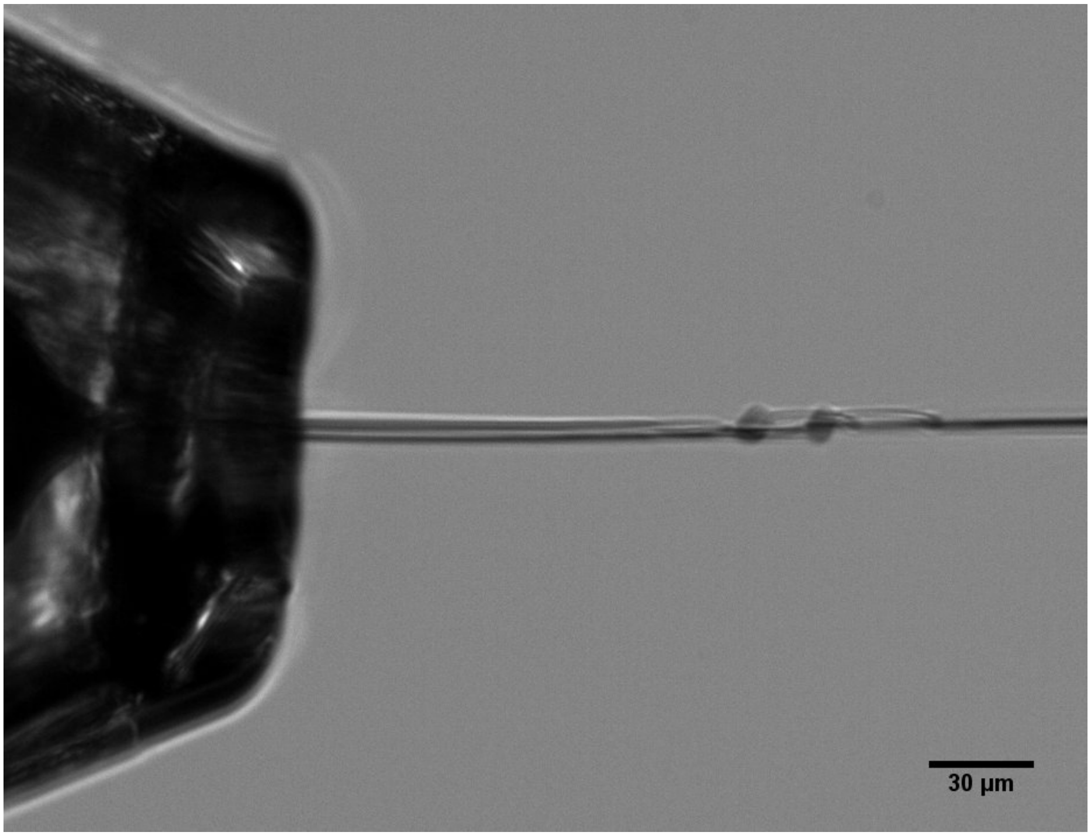

Supplementary Figure 1: Optical microscope image of a 5 µm diameter Lysozyme crystal in jet of crystal buffer at a speed of 100 m/s (condition 4 of table S1). Double-pulse illumination with a delay of 200 ns causes the crystal to appear twice in this image, enabling direct measurement of the jet speed from the distance traveled by the crystal between illumination pulses.

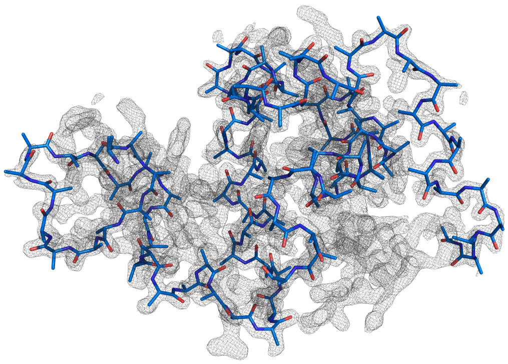

a)

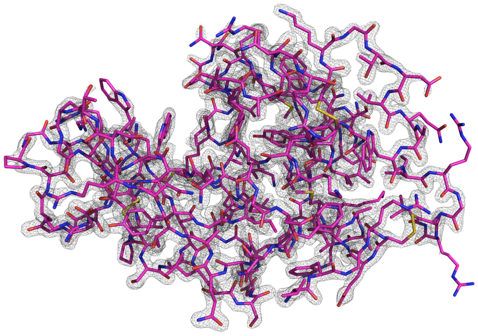

b)

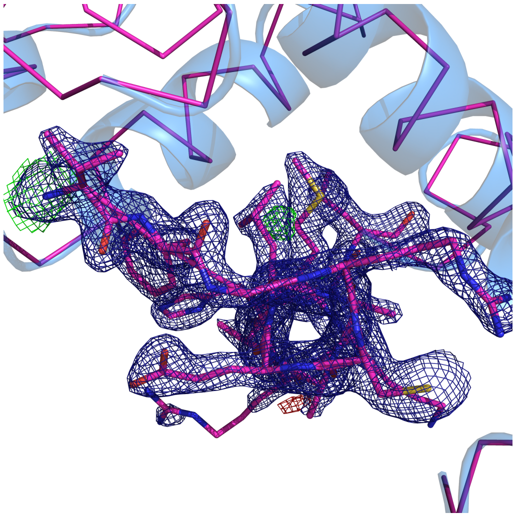

c)

Supplementary Figure 2: Autobuild map for HEWL: Rebuilding of the model using AutoBuild [1].
(a) We generated a polyAla-model of the final refined model, truncated residues 1-16 and 40-60, and then performed model building to see whether the final model could automatically be rebuilt correctly from the reflection intensities
(b) Rebuild model from starting structure in (a) using using AutoBuild [1].
(c) Highlight of the rebuilt structure (2Fc-Fc map at 1.5 sigma overlaid on Fo-Fc mapt at 3 sigma contour)

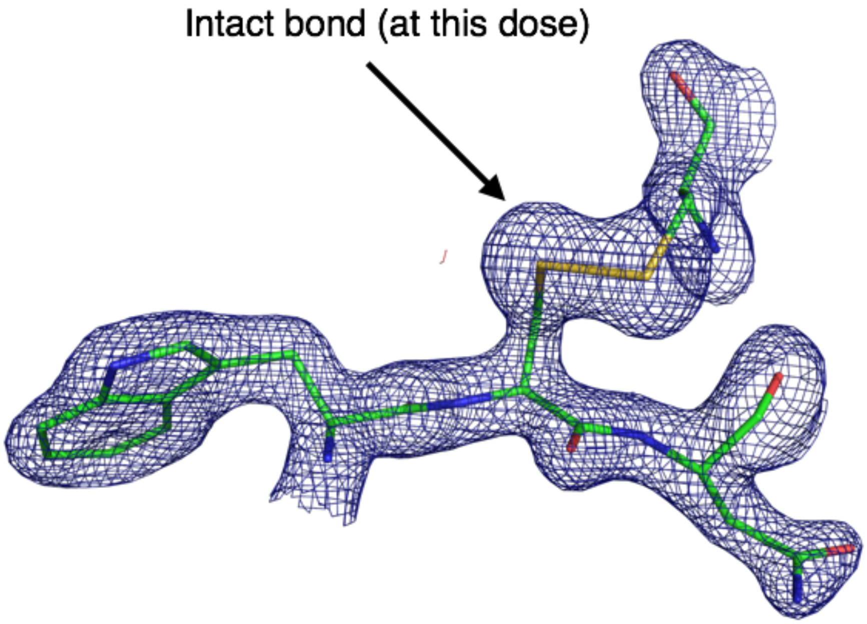

Supplementary Figure 3: No obvious signs of damage are visible at disulphide bond sites under the conditions used in this experiment.

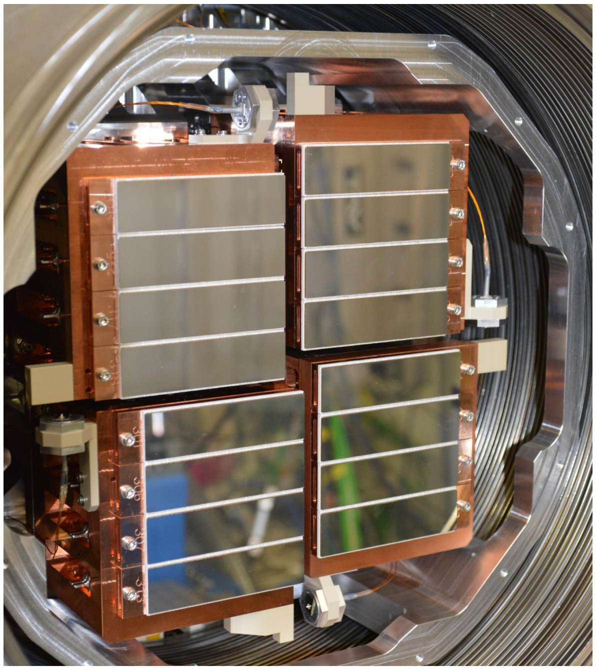

a)

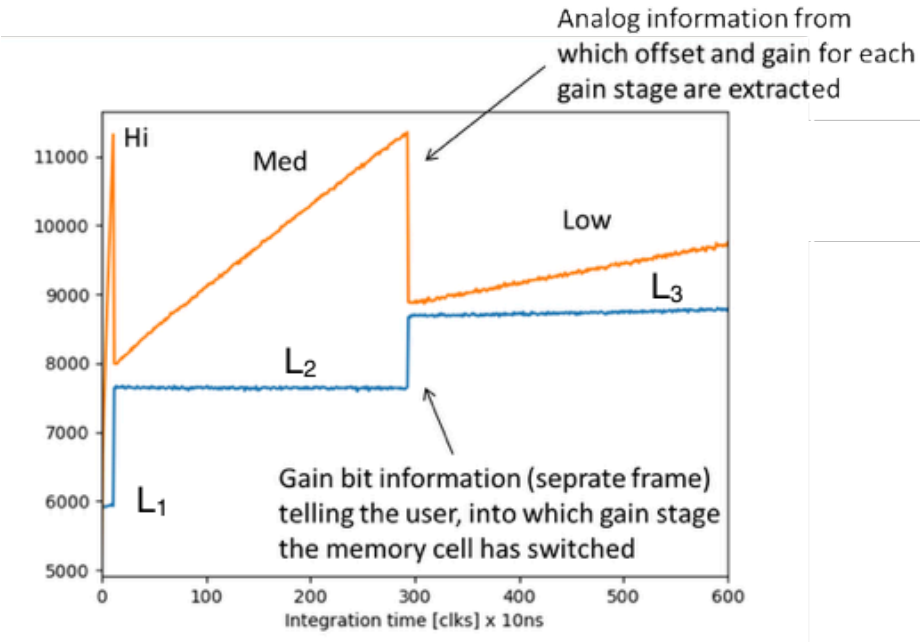

b)

Supplementary Figure 4: AGIPD detector

(a) Photograph of the active area of the AGIPD 1M detector.   used in this experiment showing the module arrangement. Note that the central gap is variable. This photograph shows the AGIPD modules driven apart. For data collection the central gap was reduced as shown in Figure 2.

(b) Illustration of the multi-stage gain switching feature of the AGIPD, in this case for constant current injection with variable integration time. All pixels start off in high gain mode, with individual pixels switching to medium gain and finally low gain modes on a per-pixel basis according to measured signal during the integration window. Detector readout is shown in the red line - gain switching enables readout to remain within range of the 14-bit Analog-to-Digital converter while dynamic range in the image is in fact much higher. A separate value, given by the blue line, indicates which gain stage is in operation for that pixel and, thus, the detector calibration values to be applied in order to recover the true signal level.

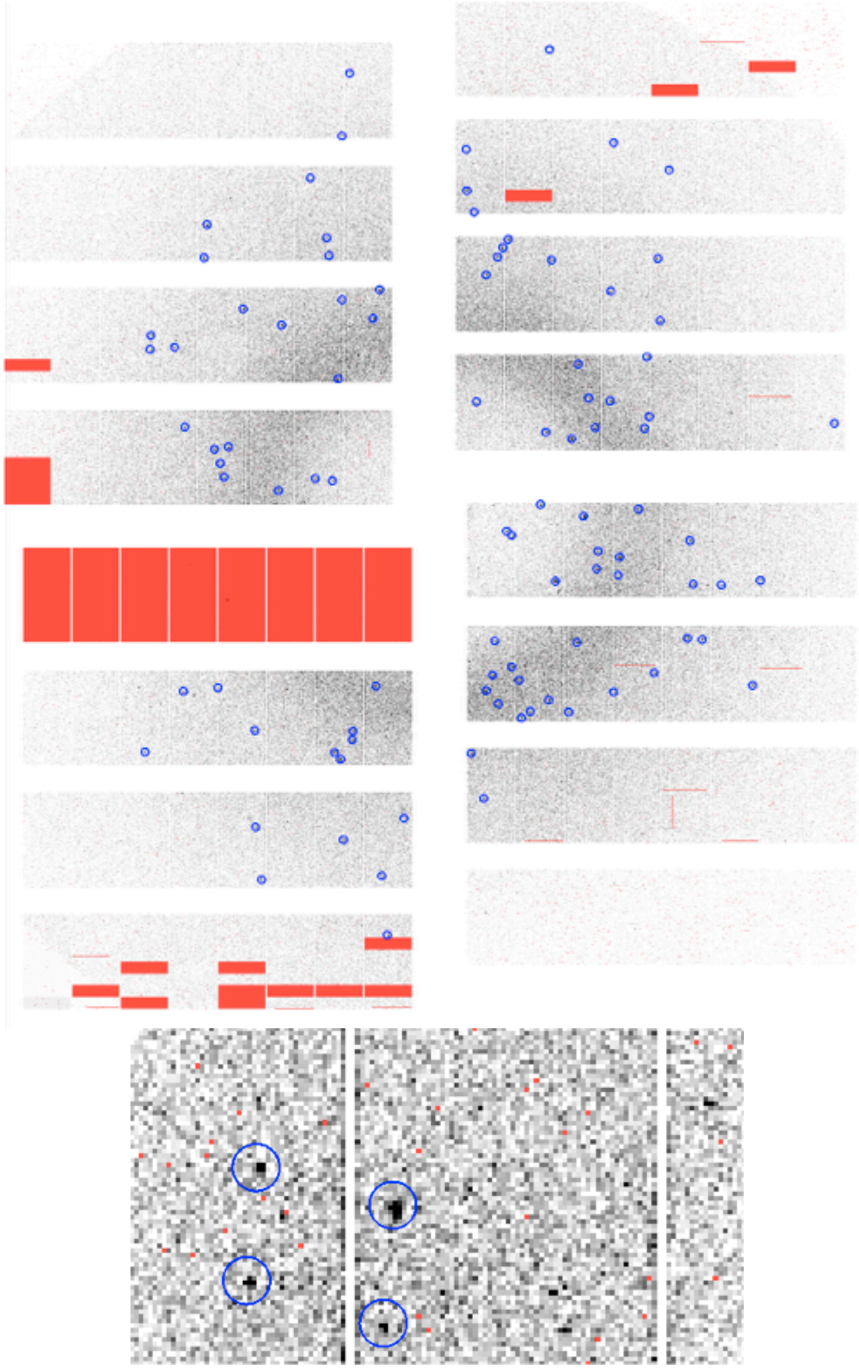

Supplementary Figure 5: Bad pixel density after application of detector calibration by Cheetah
Bad pixels and regions are shown in red after detector gain correction for a sample HEWL diffraction image: Total Number of pixels: 1 048 576; Bad pixels: 26 950 (2.57%); Bad regions*: 69 888 (6.67%); Total*: 96 838 (9.2%), the majority of which regions are in one module which had a high voltage power supply error for this experiment (since fixed) accounting for 6.25% of the overall total. This module was present for the CTX-M-14 data set. Bad pixel masks depend on memory cell and gain stage used for the image and can thus change on a frame-by-frame basis. Bad pixel density is low enough to permit integration of signal in most of the Bragg peaks.

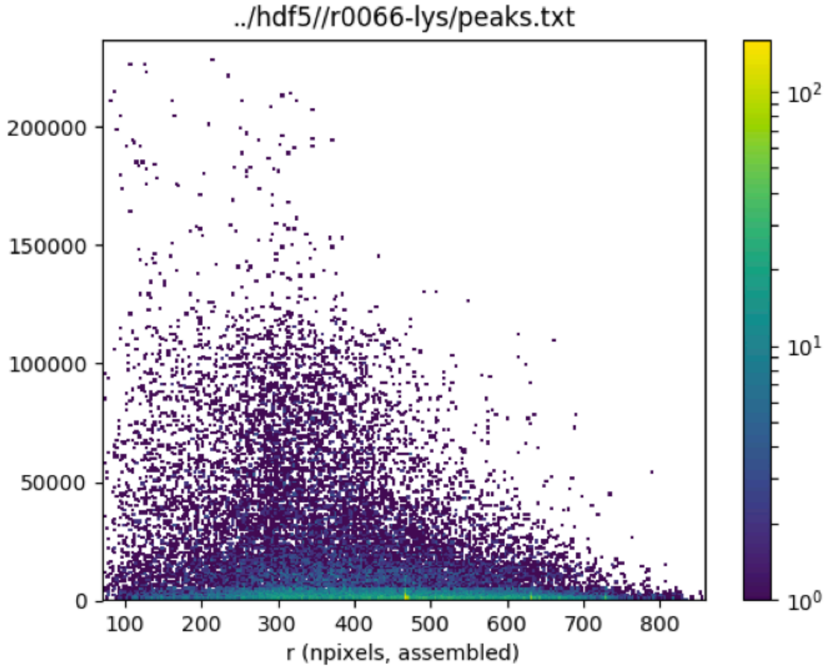

Supplementary Figure 6: Statistics on observed Bragg peaks
Distribution of maximum values in found peaks (vertical axis, in arbitrary detector units) as a function of resolution (horizontal axis measured in radius on the detector in pixels) for HEWL. Note the high dynamic range obtained through gain switching, with maximum peak intensities of over 200,000 counts in Bragg peaks observed. Note also the lack of pileup of values at detector saturation and absence of any gap in switch between low and high gain modes.

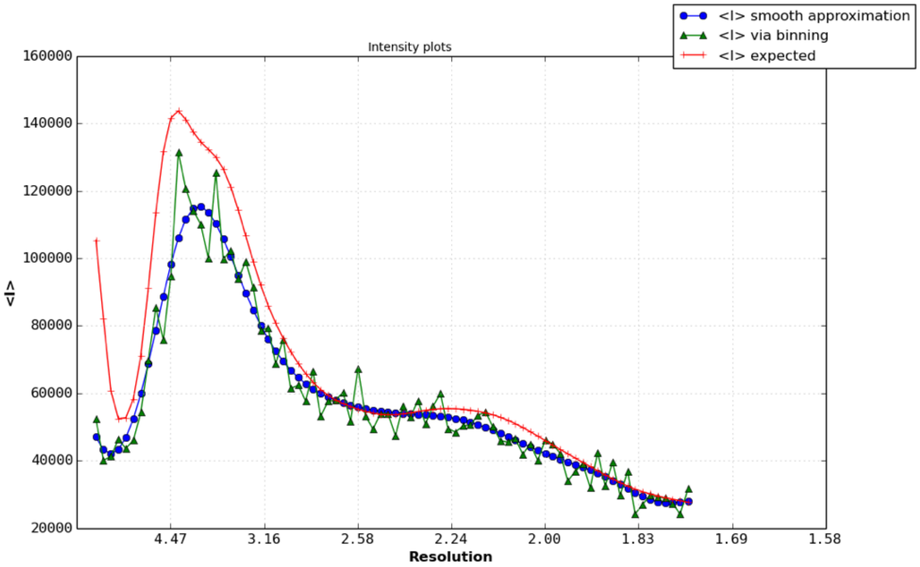

Supplementary Figure 7: Wilson plot: Wilson plot of indexed and merged data, produced by XTriage in Phenix for HEWL data.

Supplementary References

1. Terwilliger, T. C. *et al.* Iterative model building, structure refinement and density modification with the PHENIX AutoBuild wizard. *Acta Crystallogr D Biol Crystallogr* **64,** 61–69 (2008).
