# Supplementary material for: Megahertz serial crystallography
Source: Nat Commun. 2018 Oct 2;9:4025. doi: 10.1038/s41467-018-06156-7 (PMC6168542; doi:10.1038/s41467-018-06156-7)
Supplement: Supplementary file 2 — Description of Additional Supplementary Files [file 41467_2018_6156_MOESM2_ESM.pdf]

Description of Additional Supplementary Files

File Names: Supplementary Movies 1 - 4
Description: Movies of the jet breakup shown in Figure 3. Shown are jet breakups at 25 m/s (Supplementary Movie 1), 50 m/s (Supplementary Movie 2), 75 m/s (Supplementary Movie 3), and 100 m/s (Supplementary Movie 4). Jets in the range of 50-100 m/s recover in time for the next pulse, whereas slower jets of the type commonly used at LCLS explode and do not recover in time for the next XFEL pulse at MHz repetition rates. Images obtained by synchronised laser back-illumination
