# Supplementary figures and images for: Megahertz serial crystallography
Source: Nat Commun. 2018 Oct 2;9:4025. doi: 10.1038/s41467-018-06156-7 (PMC6168542; doi:10.1038/s41467-018-06156-7)

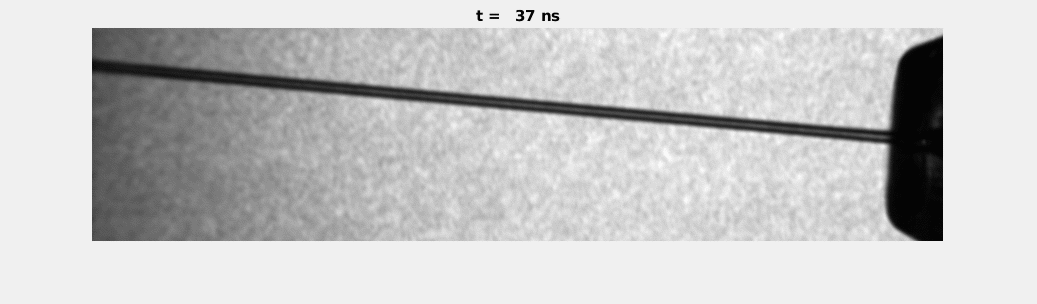

Supplement: Supplementary file 3 — Supplementary Movie 1 [file 41467_2018_6156_MOESM3_ESM.gif]

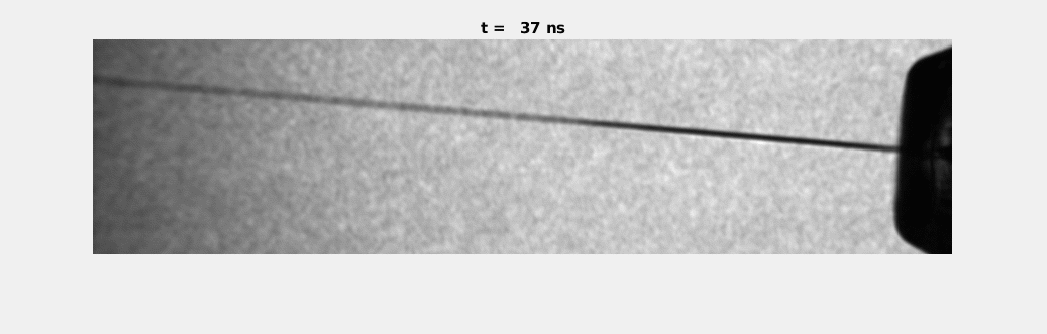

Supplement: Supplementary file 4 — Supplementary Movie 2 [file 41467_2018_6156_MOESM4_ESM.gif]

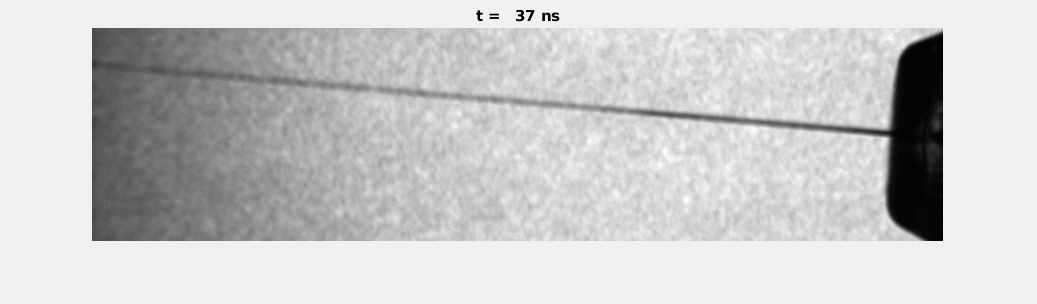

Supplement: Supplementary file 5 — Supplementary Movie 3 [file 41467_2018_6156_MOESM5_ESM.gif]

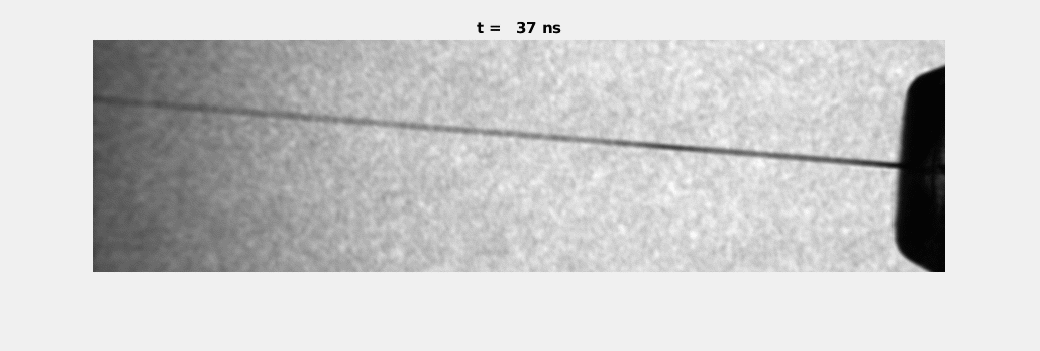

Supplement: Supplementary file 6 — Supplementary Movie 4 [file 41467_2018_6156_MOESM6_ESM.gif]
